# Transcutaneous bilirubin level to predict hyperbilirubinemia in preterm neonates

**DOI:** 10.12688/f1000research.22264.2

**Published:** 2020-09-07

**Authors:** Dewi Rahmawati, Mahendra Tri Arif Sampurna, Risa Etika, Martono Tri Utomo, Arend F. Bos

**Affiliations:** 1 Department of Pediatrics, Faculty of Medicine Universitas Airlangga, Dr. Soetomo Academic Teaching Hospital, Surabaya, Indonesia; 2 Department of Pediatrics, Beatrix Children Hospital, Universitair Medisch Centrum Groningen, Groningen, 9713 GZ, The Netherlands

**Keywords:** transcutaneous bilirubin, preterm neonates, predict, hyperbilirubinemia

## Abstract

**Background: **Hyperbilirubinemia is common in neonates, with higher prevalence among preterm neonates, which can lead to severe hyperbilirubinemia. Assessment of total serum bilirubin (TSB) and the use of a transcutaneous bilirubinometry (TcB) are existing methods that identify and predict hyperbilirubinemia. This study aimed to determine TcB cut-off values during the first day for preterm neonates to predict hyperbilirubinemia at 48 and 72 hours.

**Methods: **This cohort study was conducted at Dr. Soetomo General Hospital from September 2018 to January 2019 a total of 90 neonates born ≤35 weeks. They were divided into two groups (Group I: 1000-1500 grams; Group II: 1501-2000 grams). The bilirubin levels were measured on the sternum using TcB at the ages of 12, 24, and 72 hours. TSB measurements were taken on the third day or if the TcB level reached phototherapy threshold ± 1.24 mg/dL and if TcB showed abnormal results (Group I: 5.76-8.24 mg/dL; Group II: 8.76-11.24 mg/dL). Hyperbilirubinemia was defined as TSB ≥7 mg/dL for Group I and >10 mg/dL for Group II.

**Results: **In total, 38 Group I neonates and 48 Group II neonates were observed. Almost half of the neonates in Group I (45%) suffered from hyperbilirubinemia at the age of 48 hours, along with 46% of Group II at 72 hours. The best 24-hour-old TcB cut-off values to predict hyperbilirubinemia at 48 hours were calculated to be 4.5 mg/dL for Group I and 5.8 mg/dL for Group II. The determined 24-hour-old TcB value to predict hyperbilirubinemia at 72 hours was 5.15 mg/dL for Group II.

**Conclusion: **TcB values in the early days of life can be used as hyperbilirubinemia predictors on the following days for preterm neonates. Close monitoring should be managed for those with TcB values higher than the calculated cut-off values.

## Introduction

Hyperbilirubinemia is a common condition occurring in neonatal periods ^1^, with a prevalence of around 60% in term neonates and 80% in preterm neonates. Preterm neonates have a greater risk of severe hyperbilirubinemia, which can lead to encephalopathy ^2^. This condition is preventable if early detection and prompt treatment can be arranged and managed correctly ^1, 3, 4^.

Visual assessment is not reliable especially in the first 24–48 hours, since only 80% of jaundiced babies can be recognized visually if the bilirubin level reaches > 6 mg/dL ^5– 8^. High bilirubin levels can be dangerous, since preterm neonates have a greater risk of low bilirubin kernicterus ^2^.

Total serum bilirubin (TSB) measurement remains the gold standard for diagnosing hyperbilirubinemia. However, the drawbacks of this procedure are that it is painful, causes stress to the neonates, has a higher risk of infection, and requiresa couple of hours to obtain the results ^9– 11^.

Transcutaneous bilirubinometry (TcB) is a non-invasive procedure used to identify hyperbilirubinemia. Several studies have been conducted to validate TcB to assess whether it can be used safely. These studies found that TcB has good correlations with TSB. The use of TcB can also reduce the need for blood sampling by 41–73% ^11– 13^.

Due to the burdens of hyperbilirubinemia, its early detection and prediction are crucial. TSB or TcB is recommended to predict neonatal hyperbilirubinemia for neonates with >35 weeks of gestation ^12, 14, 15^. In a systematic review by Nagar *et al*. ^16^, most of 22 articles studied the accuracy of TcB to estimate TSB, and TcB could be used in clinical practice to reduce blood sampling. Some studies have used TcB to predict significant hyperbilirubinemia in subsequent days, but all of them recruited only late preterm and term neonates ^5, 17^. For preterm neonates, one study was already conducted using TSB measurements at 6–24 hours to predict hyperbilirubinemia in the following hours or days ^4^. As far as the researchers know, there have been no previous studies using TcB to predict subsequent, significant hyperbilirubinemia for older preterm neonates. Therefore, this study aimedto use TcB to predict hyperbilirubinemia in preterm neonates to prevent complications since visual assessment is unreliable.

## Methods

### Study background and ethical approval

This cohort study was conducted in the neonatal intensive care unit at Dr Soetomo General Hospital for five months (September 2018–January 2019). This study was approved by Dr. Soetomo General Hospital Surabaya Ethics Committee (No. 0586/KEPK/Ix/2018). Parents signed the informed consent form after they understood the information. The study size retrieved in this research used purposive sampling with inclusion and exclusion criteria (a flow diagram is available as *Extended data*) ^18, 19^ during the research period. The sample size was estimated by applying Hulley *et al*.’s ^20^ formulation, with a confidence interval of 95%, a coefficient correlation of 0.84 and a standard deviation of 1.8 ^4^. Therefore, the minimum sample size of 20 was applied for each group, classified by infants’ body weights. Staying in line with the minimum sample size, the samples were expanded up to 45 infants for each group, with a total sample of 90 infants. However, four datasets were excluded due to missing TSB measurement.

Race and thickness of the skin tissue’s melanin layer were taken into account as confounding variables, and as variables able to change the outcomes of others. Therefore, to control for study bias, the subjects addressed for this study were those with similar ethnic backgrounds: Malay Mongoloid.

### Participant eligibility

The inclusion criteria were: 1) birth at ≤35 weeks of gestational age with a birth weight < 2000 g, and 2) parental consent given by signing a form. The exclusion criteria were: 1) being diagnosed with hyperbilirubinemia at the age of 12 hours, 2) having any major congenital anomaly, or 3) being discharged from hospital at less than three days old. Neonates who received phototherapy before the observation was complete, missed TSB, or voluntarily resigned from this study were excluded from the study. The recruited subjects were divided into two groups: neonates with birth weights of 1001-1500 g (Group I) and 1501-2000 g (Group II).

### Variables

The bilirubin level of each neonate was measured on the sternum by TcB (Dräger® Jaundice Meter 105) at 12 hours, 24 hours, and 72 hours with ±3 hours tolerance (the TcB measurement could be taken within three hours before/after the exact time). The TSB measurement was taken for each neonate at the age of three days or if the TcB bilirubin level was ≥5.76 (7–1.24) mg/dL for Group I and TcB ≥8.76 (10–1.24) mg/dL for Group II and it had to be taken within six hours before or after the TcB measurement (assumption of TcB standard deviation being ±1.24 mg/dL). The TSB measurement also had to be taken if the TcB measurement showed abnormal results. Hyperbilirubinemia was defined as TSB ≥7 mg/dL for preterm neonates with birth weights of 1000–1500 g and TSB >10 mg/dL for preterm neonates with birth weights of 1501–2000 g as suggested by the Kaplan *et al.,* in Martin’s Neonatal-Perinatal Medicine (2011). ^21^ TSB was measured in the central laboratory using SIEMENS Dimension® with a modified Doumas ^22^ reference method, which is a modification of the diazo method described by Jendrassik and Grof in 1938 ^23^. Internal calibration was completed daily, with a quality control printout. The Indonesian External Quality Assurance Service performed external quality control.

### Statistical analysis

The data was analyzed by Microsoft Office Excel, IBM SPSS Statistics Version 21. Receiver operating characteristic (ROC) curve analysis was performed to determined the TcB level cut-off point to predict hyperbilirubinemia at the age of 48 and 72 hoursThe specificity, sensitivity, positive predicted value (PPV), negative predicted value (NPV), and likelihood ratio were calculated.

## Results

There were 90 preterm neonates recruited for this study, 40 of whom weighed 1000–1500 grams (Group I) and 50 of whom weighed 1501–2000 grams (Group II). Only 38 neonates in Group I and 48 neonates of Group II were observed until the end of the study. Four neonates were excluded from the study due to missing TSB results.

**Figure 1.**
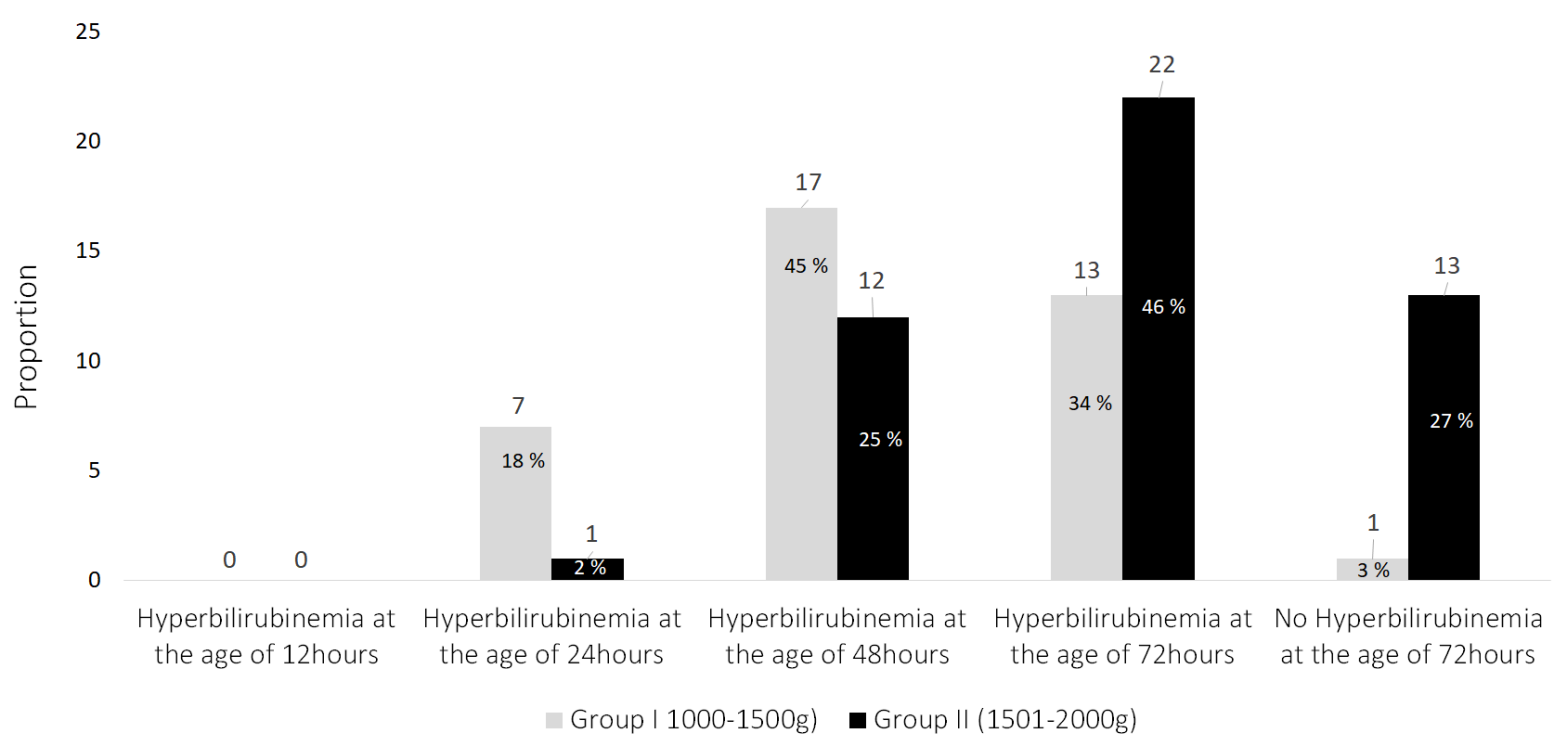
The proportionof hyperbilirubinemia in preterm neonates.

Maternal and neonatal characteristics are shown in Table 1. For Group I, the mean gestational age of Group I was 32.29 ± 1.84 weeks, with a mean birth weight of 1273.68 ± 177.34 g. Meanwhile, for Group II the mean gestational age was 33.69 ± 1.26 weeks, witha mean birth weight of 1792.70 ± 145.86 g. Based on the risk factors of ABO-incompatibility, one subject of Group I who suffered hyperbilirubinemia at the age of 48 hours. Meanwhile, two subjects in Group II suffered hyperbilirubinemia at the age of 48 hours and another two subjects at the age of 72 hours. At the end of the observation, the maximum bilirubin level was 15.2 mg/dl for Group I and 16.33 mg/dL for Group II. Most neonates of Group I (45%) suffered hyperbilirubinemia at the age of 48 hours, while most neonates of group II (46%) at the age of 72 hours ( Figure 1). The TSB means in Group I at the ages of 24, 48, and 72 hours were 7.9 mg/dL, 9.16 mg/dL, and 9.3 mg/dL respectively, and 11.01 mg/dL, 10.23 mg/dL, and 11.04 mg/dL respectively, in Group II.

**Table 1.** Maternal and neonatal characteristics of subjects.

Maternal characteristics	Group I (n=38) n(%)	Group II (n=48) n(%)
Gestational Age (weeks) (mean ±SD)	32.29 ± 1.84	33.69 ± 1.26
Mode of delivery - spontaneous - c-section - vacuum	11(29) 26(68) 1(3)	9(19) 38(80) 1(1)
Maternal Blood Type A B O AB	7(18.4) 14(36.80) 16(42.10) 1(2.70)	12(25) 10(20.80) 19(39.60) 7(14.60)
Neonatal characteristics	Group I (n=38) n (%)	Group II (n=48) n (%)
Birth Weight (g) (mean ±SD)	1273.68 ± 177.34	1792.70 ± 145.86
Hematocrit (%) (mean ±SD)	48.04 ± 10.47	46.99± 8.48
Gender Male Female	19 (50) 19 (50)	30(62.50) 18(37.50)
Neonatal blood-type A B O AB	4(10.50) 11(28.90) 21(55.30) 2(5.30)	9(18.8) 10(20.8) 24(50) 5(10.4)

^*^Descriptive analysis was used. Maternal and neonatal rhesus were positive.

### Using TcB bilirubin levels to predict hyperbilirubinemia at the age of 48 hours in Group I preterm neonates

**Figure 2.**
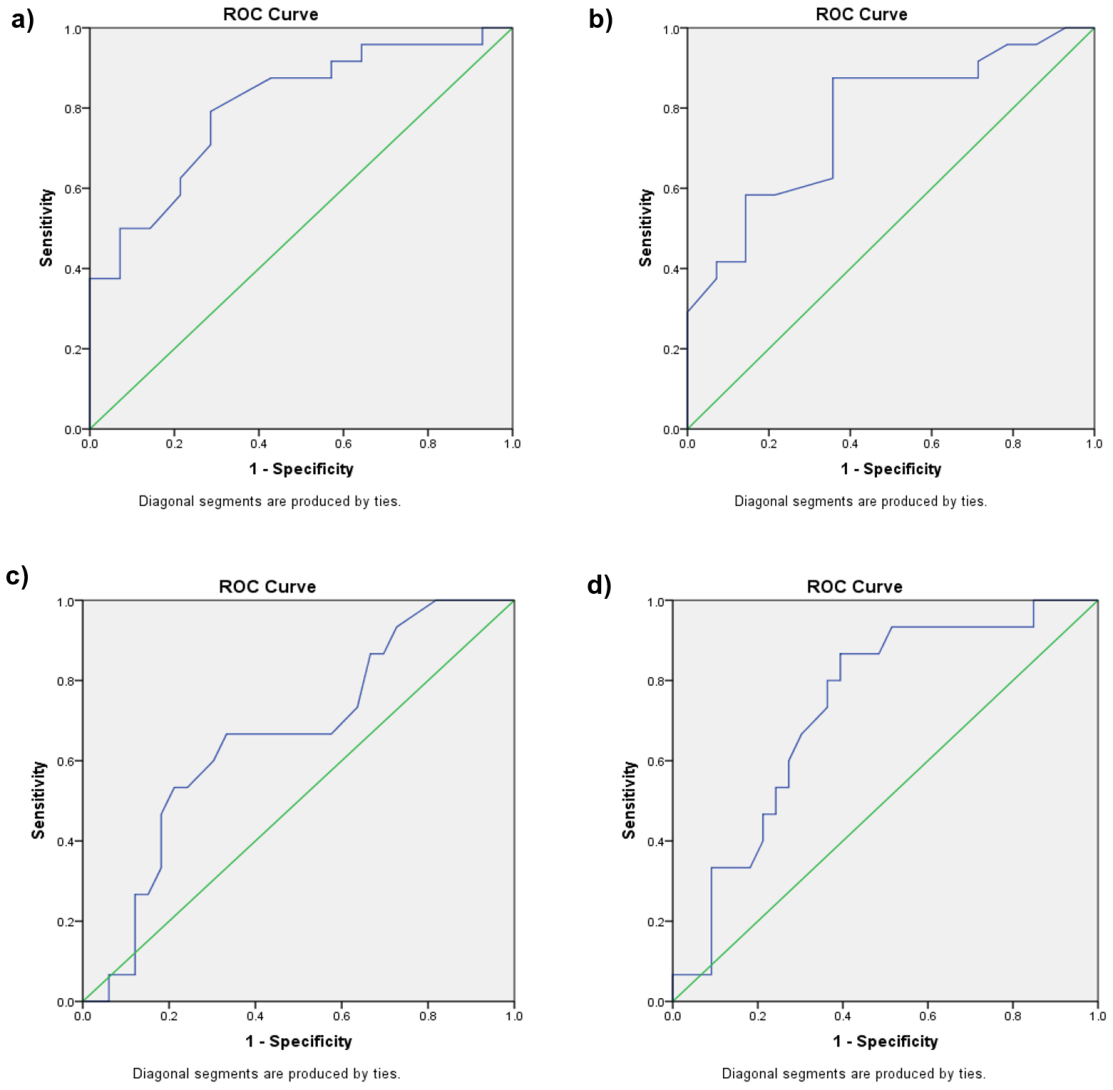
Transcutaneous bilirubinometry (TcB) level to predict hyperbilirubinemia at the age of 48 hours. ( **a**) Receiver operating characteristic (ROC) curve for TcB at the age of 12 hours to predict hyperbilirubinemia at the age of 48 hours for Group I. ( **b**) ROC curve for TcB at the age of 24 hours to predict hyperbilirubinemia at the age of 48 hours for Group I. ( **c**) ROC curve for TcB at the age of 12 hours to predict hyperbilirubinemia at the age of 48 hours for Group II. ( **d**) ROC curve for TcB at the age of 24 hours to predict hyperbilirubinemia at the age of 48 hours for Group II.

A ROC curve was constructed to determine a hyperbilirubinemia threshold based on the data collected. For Group I, the area under the curve (AUC) of the TcB bilirubin level at the age of 12 hours to predict hyperbilirubinemia at the age of 48 hours was 0.804 (p = 0.002), with a cut-off point of 2.35 mg/dL (sensitivity: 79.20%;specificity: 71.40%). For TcB bilirubin levels at the age of 24 hours to predict hyperbilirubinemia at the age of 48 hours, the AUC was 0.771 (p = 0.06), with a cut-off point of 4.50mg/dL (sensitivity: 87.50%; specificity: 64.26%) ( Figure 2a, Figure 2b, Table 2).

**Table 2.** Transcutaneous bilirubinometry (TcB) bilirubin level cut-off point to predict hyperbilirubinemia at the age of 48 hours for Group I.

TcB level cut-off (mg/dL)		Group I				
		Sn (%)	Sp (%)	PPV (%)	NPV (%)	LR
12 hours old	2.35	79.2	71.4	82.60	66.67	2.78
24 hours old	4.5	87.5	64.3	80.77	64.26	2.45

^†^Receiver operative characteristic curve analysis was used. Sn: sensitivity; Sp: specificity; PPV: positive predictive value; NPV: negative predictive value; LR: likelihood ratio.

### Using TcB bilirubin levels to predict hyperbilirubinemia at the age of 48 hours in Group II preterm neonates

The AUC of TcB bilirubin levels at the age of 12 hours to predict hyperbilirubinemia at the age of 48 hours for Group II was 0.658 (p = 0.083), with a cut-off point of 3.05 mg/dL (sensitivity: 66.7%; specificity: 66.7%). The AUC of TcB bilirubin levels at the age of 24 hours was 0.732 (p = 0.011), with a cut-off point of 5.80 mg/dL (sensitivity: 80%; specificity: 63.6%). ( Figure 2c, Figure 2d and Table 3).

**Table 3.** TcB bilirubin level cut-off point to predict hyperbilirubinemia at the age of 48 hours for Group II.

TcB level cut-off (mg/dL)		Group II				
		Sn (%)	Sp (%)	PPV (%)	NPV (%)	LR
12 hours old	3.05	66.7	66.7	47.62	81.48	2.00
24 hours old	5.85	80	63.6	50.00	87.50	2.19

^†^Receiver operative characteristic curve analysis was used. Sn: sensitivity; Sp: specificity; PPV: positive predictive value; NPV: negative predictive value; LR: likelihood ratio.

### Using TcB bilirubin levels to predict hyperbilirubinemia at the age of 72 hours in Group I preterm neonates

The TcB bilirubin levels of Group I at the ages of 12, 24, and 48 hours to predict hyperbilirubinemia at the age of 72 hours showed a very weak AUCs, which were 0.243 (p = 0.386), 0.297 (p = 0.494), 0.500 (p = 1.000), respectively; therefore, no cut-off point could be determined.

### Using TcB bilirubin levels to predict hyperbilirubinemia at the age of 72 hours in Group II preterm neonates

**Figure 3.**
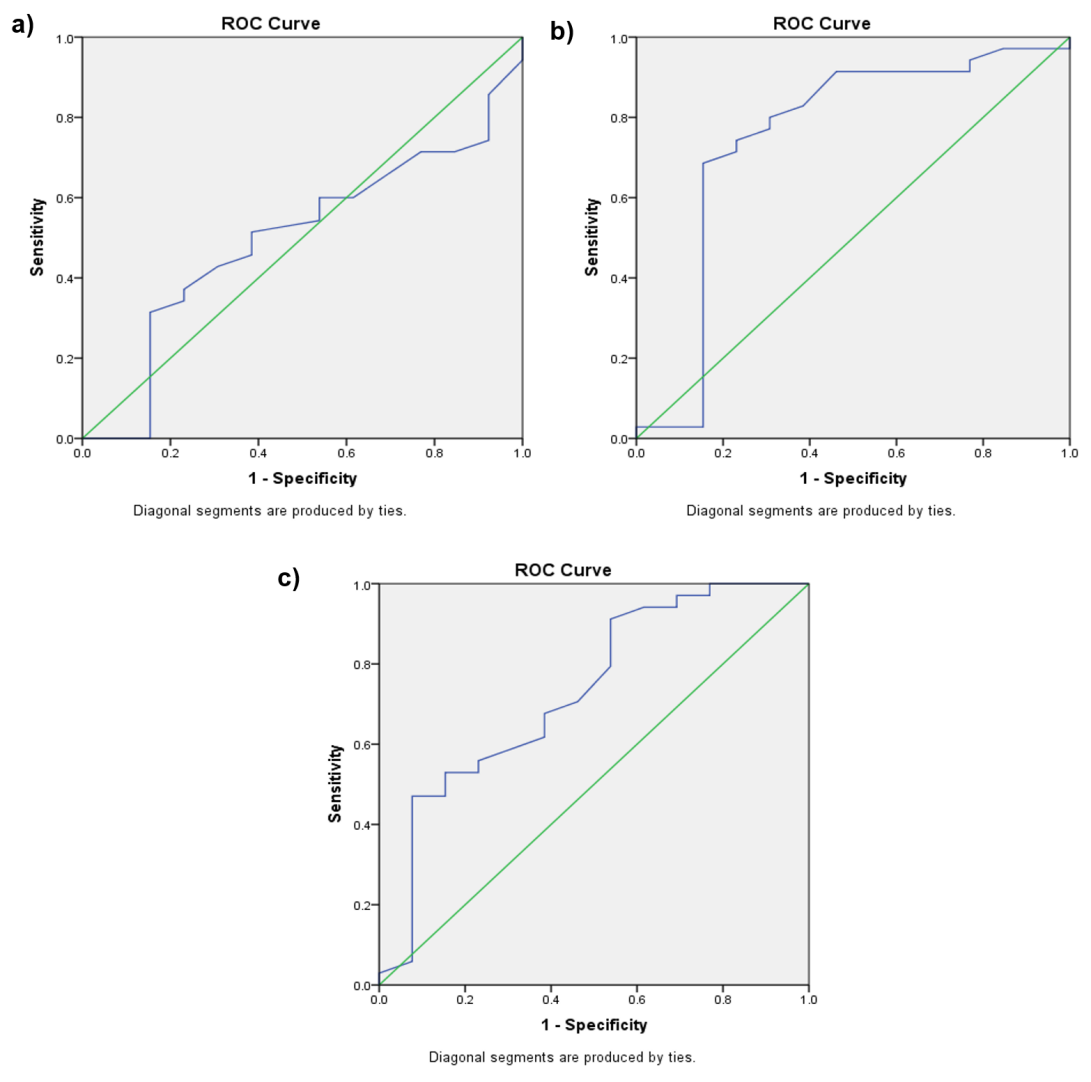
Transcutaneous bilirubinometry (TcB) level to predict hyperbilirubinemia at the age of 72 hours. ( **a**) Receiver operating characteristic (ROC) curve for TcB at the age of 12 hours to predict hyperbilirubinemia at the age of 72 hours for Group II. ( **b**) ROC curve for TcB at the age of 24 hours to predict hyperbilirubinemia at the age of 72 hours for Group II. ( **c**) ROC curve for TcB at the age of 48 hours to predict hyperbilirubinemia at the age of 72 hours for Group II.

Using the TcB bilirubin levels of Group II at the age of 12 hours to predict hyperbilirubinemia at the age of 72 hours showed a weak AUC (0.499, p = 0.991) with a cut-off point of 2.65 mg/dL (sensitivity 60% and specificity 46%). At the age of 24 hours, the TcB AUC was 0.751 (p = 0.008), with a cut-off point of 5.15 mg/dL (sensitivity: 74.3%; specificity: 76.9%). Meanwhile, at the age of 48 hours the TcB AUC was 0.731 (p = 0.015), with a cut-off point of 8.65 mg/dL (sensitivity: 67.6%; specificity: 61%) ( Figures 3a–c and Table 4).

**Table 4.** TcB bilirubin level cut-off point to predict hyperbilirubinemia at the age of 72 hours for Group II.

TcB level cut-off (mg/dL)		Group II				
		Sn (%)	Sp (%)	PPV (%)	NPV (%)	LR
12 hours old	2.65	60	46	75.00	30.00	1.11
24 hours old	5.15	74.3	76.9	89.66	52.63	3.22
48 hours old	8.65	67.6	61	82.75	42.10	1.78

^†^Receiver operative characteristic curve analysis was used. Sn: sensitivity; Sp: specificity; PPV: positive predictive value; NPV: negative predictive value; LR: likelihood ratio.

## Discussion

This study determined a TcB cut-off value of 4.5 mg/dL at the age of 24 hours for Group I (1000–1500 grams) and 5.8 mg/dL for Group II (1501–2000 grams) as predictive of hyperbilirubinemia at 48 hours. This study could not determine a TcB cut-off value to predict hyperbilirubinemia at the age of 72 hours for Group I as a result of weak correlation. The TcB cut-off value of 5.15 mg/dL at the age of 24 hours was determined as the best predictor for hyperbilirubinemia at the age of 72 hours in Group II. This cut-off level was established with a sensitivity value ranging from 74.3% to 87.5% at 24 hours after birth. Similar studies have already been conducted, but those studies recruited only late preterm neonates. Lavanya *et al.* found that TcB values measured in the first 24–48 hours of life can predict hyperbilirubinemia at more than 48 hours ^24^. Bansal *et al.* determined that TcB values > 4.6 mg/dL at 12–24 hours (sensitivity: 83.09%; specificity: 87.37%; PPV: 90.4%; NPV: 78.3%) and > 7.4 mg/dL at the age of 24–48 hours (sensitivity: 93.55%; specificity: 82.11%; PPV: 81.69%; NPV: 95.35%) are predictors for hyperbilirubinemia in the first 48 hours of life ^5^. Other studies used TSB values to predict hyperbilirubinemia in the following days. Mayer recruited preterm neonates weighing 1000–1500 grams and determined a capillary TSB value of 3.55 mg/dL at the age of 12 hours as the best predictor of significant hyperbilirubinemia (sensitivity: 94.4%; PPV: 98.1%; NPV: 40%) ^4^.

Most neonates recruited in this study suffered hyperbilirubinemia before the age of 72 hours. This study also showed that smaller babies suffered peak incidence earlier (at 48 hours) than larger babies (at 72 hours). It shows that that higher birth weight has opportunity to be hyperbilirubinemia later than the lower birthweight. The lower birthweight has lower threshold bilirubin, because they have higher chance to become encephalopathy in lower bilirubin level (high risk infants). The younger gestational age and lower birth weight, lead to a higher prevalence of infants developing hyperbilirubinemia. It is a result of excessive neonatal red cell hepatic and immaturity of the gastrointestinal system. Prematurely delivered infants have a likelihood of slower maturation of hepatic bilirubin uptake and conjugation ^25, 26^. Hyperbilirubinemia is more prevalent in preterm neonates ^4, 5, 24^) and is usually more severe and has a longer duration compared to that in term neonates ^4^. This is caused by increased bilirubin production, decreased bilirubin excretion, increased enterohepatic circulation, lower albumin levels and a weak albumin-bilirubin bond ^10, 27^. Early detection of hyperbilirubinemia decreases its mortality and morbidity, and the need for reliable methods to predict hyperbilirubinemia is crucial. The use of a non-invasive procedure, like TcB, in the first 6–24 hours of life is recommended as a marker of bilirubin production ^28^ and it can decrease the need for blood sampling ^29^.

In Group I at the ages of 24, 48, and 72 hours, the TSB mean values were 7.9, 9.16, and 9.3 mg/dL, respectively; in Group II, these values were 11.01, 10.23, and 11.04 mg/dL, respectively. This is similar to a previous study that found that TSB mean values in the first five days of preterm neonates were 10–12 mg/dL ^21^. However, this study found that some preterm neonates reached a TSB value >15 mg/dL in the first 72 hours. Preterm neonates can have high bilirubin levels in the first days of life, which can lead to hyperbilirubinemia complications if not recognized and treated properly. The previous study conducted by Bhutani *et al*., also indicated that neonates who suffered from hyperbilirubinemia in the following days had higher percentiles on the first day of life ^30^. Therefore, the American Academy of Pediatrics recommends routine checks of TSB or TcB along with risk factor assessments in the first days of life ^14^.

To the knowledge of the researchers, this was the first study conducted to predict significant hyperbilirubinemia in preterm neonates weighing 1000–2000 grams using TcB. A limitation of the study was that it could not determine TcB cut-off values to predict hyperbilirubinemia at the age of 72 hours for preterm neonates weighing 1000–1500 grams due to a lack of subjects able to complete the study, since most had already developed significant hyperbilirubinemia by this time. The mothers’ rhesus blood groups and the babies’ G6PD levels were not obtained. Hopefully, future similar studies will be able to recruit larger populations.

## Conclusion

TcB values in the early days of life can be used as a predictor of hyperbilirubinemia in the following days for preterm neonates. Preterm neonates can have high bilirubin levels in the first few days of their lives. Therefore, daily TcB measurement is important for early identification of hyperbilirubinemia, especially to prevent complications in certain more vulnerable preterm neonates. Close monitoring should be arranged for those who have TcB values higher than the cut-off values.

## Data availability

### Underlying data

Figshare: Datasheet TcB and TSB - Group I. https://doi.org/10.6084/m9.figshare.11948490.v1 ^31^.

This project contains data gathered for neonates in Group I (those 1000–1500 grams).

Figshare: TcB Level and TSB-MTA - Group II. https://doi.org/10.6084/m9.figshare.11948529.v1 ^32^.

This project contains data gathered for neonates in Group II (those 1500–2000 grams).

### Extended data

Figshare: Supplemental File - Flow Chart Study of TcB and TSB. https://doi.org/10.6084/m9.figshare.12017586.v1 ^19^.

This project contains a study flow diagram.

### Reporting guidelines

Figshare: STROBE checklist for ‘Transcutaneous bilirubin level to predict hyperbilirubinemia in preterm neonates’. https://doi.org/10.6084/m9.figshare.11991672.v2 ^18^.

Data are available under the terms of the Creative Commons Attribution 4.0 International license (CC-BY 4.0).
