# Supplementary material for: Transcutaneous bilirubin level to predict hyperbilirubinemia in preterm neonates
Source: F1000Res. 2020 Sep 7;9:300. Originally published 2020 Apr 28. [Version 2] doi: 10.12688/f1000research.22264.2 (PMC7499403; doi:10.12688/f1000research.22264.2)
Supplement: Peer review history

# Peer review: Reviewer response for version 2

**DOI:** 10.5256/f1000research.27678.r70954

**Published:** 2020-09-17

**Authors:** Tina M. Slusher

**Affiliations:** 1 Department of Pediatrics, University of Minnesota, Minneapolis, MN, USA

Language in places is still awkward and could be improved on but message is clearer and concerns have been addressed.

Is the work clearly and accurately presented and does it cite the current literature?

Partly

If applicable, is the statistical analysis and its interpretation appropriate?

Partly

Are all the source data underlying the results available to ensure full reproducibility?

Partly

Is the study design appropriate and is the work technically sound?

Partly

Are the conclusions drawn adequately supported by the results?

Partly

Are sufficient details of methods and analysis provided to allow replication by others?

Yes

Reviewer Expertise:

Pediatric Global Health, Neonatal Hyperbilirubinemia, Pediatric Critical Care

I confirm that I have read this submission and believe that I have an appropriate level of expertise to confirm that it is of an acceptable scientific standard.

# Peer review: Reviewer response for version 2

**DOI:** 10.5256/f1000research.27678.r70951

**Published:** 2020-09-08

**Authors:** Claudio Tiribelli

**Affiliations:** 1 Italian Liver Foundation, Trieste, Italy

Authors have addressed my concerns.

Is the work clearly and accurately presented and does it cite the current literature?

No

If applicable, is the statistical analysis and its interpretation appropriate?

Partly

Are all the source data underlying the results available to ensure full reproducibility?

No

Is the study design appropriate and is the work technically sound?

No

Are the conclusions drawn adequately supported by the results?

Partly

Are sufficient details of methods and analysis provided to allow replication by others?

No

Reviewer Expertise:

Bilirubin, Jaundice

I confirm that I have read this submission and believe that I have an appropriate level of expertise to confirm that it is of an acceptable scientific standard.

# Peer review: Reviewer response for version 1

**DOI:** 10.5256/f1000research.24559.r62845

**Published:** 2020-05-21

**Authors:** Tina M. Slusher

**Affiliations:** 1 Department of Pediatrics, University of Minnesota, Minneapolis, MN, USA

This study is an interesting study looking at the correlation between transcutaneous bilirubin (TcB) and serum bilirubin and the predictive value of subsequent clinically significant hyperbilirubinemia in neonates. However, there are some problems with the study that need to be addressed. 

1. They authors state that TcB has not been used in premature infants with the exception of late preterms. However, in a systematic review by Nager *et al.* most of the 22 articles they mention include very preterm infants.  Perhaps the authors need to mean to say in their population but this needs to be clarified.
2. Each country does indeed need to develop their own criteria for diagnosis and treatment levels of hyperbilirubinemia based on risk in their environment, treatments available and other specifics related to their own country.  However, authors do need to tell us where there cutoffs for each group came from---are the in country normal, standard cutoffs for their hospital or region or how were they selected.
3. Numbers too small to extrapolate widely to Indonesia or beyond.
4. Rhesus of mothers and infants missing; G6PD status missing if not done state that.
5. Although not a major problem or limitation this article could be improved by having a native English speaker read and make minor edits throughout.

Is the work clearly and accurately presented and does it cite the current literature?

Partly

If applicable, is the statistical analysis and its interpretation appropriate?

Partly

Are all the source data underlying the results available to ensure full reproducibility?

Partly

Is the study design appropriate and is the work technically sound?

Partly

Are the conclusions drawn adequately supported by the results?

Partly

Are sufficient details of methods and analysis provided to allow replication by others?

Yes

Reviewer Expertise:

Pediatric Global Health, Neonatal Hyperbilirubinemia, Pediatric Critical Care

I confirm that I have read this submission and believe that I have an appropriate level of expertise to confirm that it is of an acceptable scientific standard, however I have significant reservations, as outlined above.

# Response

**Published:** 2020-06-25

**Authors:** Mahendra Tri Arif Sampurna

**Affiliations:** Faculty of Medicine Universitas Airlangga, Indonesia

**1). Reviewer's comments:**

 This study is an interesting study looking at the correlation between transcutaneous bilirubin (TcB) and serum bilirubin and the predictive value of subsequent clinically significant hyperbilirubinemia in neonates. However, there are some problems with the study that need to be addressed.

 The authors state that TcB has not been used in premature infants with the exception of late preterms. However, in a systematic review by Nager *et al.* most of the 22 articles they mention include very preterm infants.  Perhaps the authors need to mean to say in their population but this needs to be clarified.

**Author's response:**

 Dear Reviewer,

 Thank you for your comments.

   

 Yes, indeed you are right, thanks for your suggestion. What we meant here is similar with the study by Mayer (Mayer I, Gursoy T, Hayran M, Ovalı F. Value of twelfth-hour bilirubin level in predicting significant hyperbilirubinemia in preterms Infants. J Clin Med Res. 2014;6(3):190–6) but they used TSB to predict significant hyperbilirubinemia later on. Practice in our hospital so far is using Kramer and few TSB measurement to start phototherapy. So, we give another alternative which is non-invasive, easy to use, and applicable in our setting in Indonesia.  

 We added in our introduction as :

 In a systematic review by Nagar et al. ^16^, most of 22 articles studied the accuracy of TcB to estimate TSB, and TcB could be used in clinical practice to reduce blood sampling. Some studies have used TcB to predict significant hyperbilirubinemia in subsequent days, but all of them recruited only late preterm and term neonates ^5,17^ . For preterm neonates, one study was already conducted using TSB measurements at 6- 24 hours to predict hyperbilirubinemia in the following hours or days ^4^ . As far as the researchers know, there have been no previous studies using TcB to predict subsequent, significant hyperbilirubinemia for older preterm neonates. Therefore, this study aimed to use TcB to predict hyperbilirubinemia in preterm neonates to prevent complications since visual assessment is unreliable.

**2) Reviewer's comment: **Each country does indeed need to develop their own criteria for diagnosis and treatment levels of hyperbilirubinemia based on risk in their environment, treatments available and other specifics related to their own country.  However, authors do need to tell us where there cutoffs for each group came from---are the in country normal, standard cutoffs for their hospital or region or how were they selected

**Author's response:**

 Thank you for your comments.

 We added in our method à variables section as follows:

 The bilirubin level of each neonate was measured on the sternum by TcB (Dräger® Jaundice Meter 105) at 12 hours, 24 hours, and 72 hours with ±3 hours tolerance (the TcB measurement could be taken within three hours before/after the exact time). The TSB measurement was taken for each neonate at the age of three days or if the TcB bilirubin level was ≥5.76 (7-1.24)  mg/dL for Group I and TcB ≥8.76 (10-1.24) mg/dL for Group II and it had to be taken within six hours before or after the TcB measurement (assumption of TcB  standard deviation being ±1.24 mg/dL). The TSB measurement also had to be taken if the TcB measurement showed abnormal results. Hyperbilirubinemia was defined as TSB ≥7 mg/dL for preterm neonates with birth weights of 1000–1500 g and TSB >10 mg/dL for preterm neonates with birth weights of 1501–2000 g as suggested by the Kaplan et al., in Martin’s Neonatal-Perinatal Medicine (2011). ^21^

****

**3). Reviewer's comment:**

 Numbers too small to extrapolate widely to Indonesia or beyond.

**Author's response:**

 Dear Reviewer,

 This is the study limitation and has been addressed in discussion section.

 We added in the discussion section in the last paragraph:

 A limitation of the study was that it could not determine TcB cut-off values to predict hyperbilirubinemia at the age of 72 hours for preterm neonates weighing 1000–1500 grams due to a lack of subjects able to complete the study, since most had already developed significant hyperbilirubinemia by this time.

**4). Reviewer's comment:**

 Rhesus of mothers and infants missing; G6PD status missing if not done state that.

**Author's response:**

 Dear Reviewer,

 Thank you for your comments.

 We even hardly measured Blood Group ABO and Rhesus of the mother and babies since the babies are not yellow. We have not used it as screening program. G6PD measurement is not affordable for our majority of citizen. We need to give consent first when the bilirubin is not yet decrease and rise again after intensive phototherapy, or there is sign of haemolysis 

 Thank you for your suggestion. Indeed, this is also our limitation that we added in the discussion section on the last paragraph

 To the knowledge of the researchers, this was the first study conducted to predict significant hyperbilirubinemia in preterm neonates weighing 1000–2000 grams using TcB. A limitation of the study was that it could not determine TcB cut-off values to predict hyperbilirubinemia at the age of 72 hours for preterm neonates weighing 1000–1500 grams due to a lack of subjects able to complete the study, since most had already developed significant hyperbilirubinemia by this time. The mothers’ rhesus blood groups and the babies’ G6PD levels were not obtained. Hopefully, future similar studies will be able to recruit larger populations.

**5) Reviewer's comment:**

 Although not a major problem or limitation this article could be improved by having a native English speaker read and make minor edits throughout

**Author's response:**

 Dear Reviewer,

 Thank you for your comments

 We have already proofread this manuscript by Proofreading Service.

# Peer review: Reviewer response for version 1

**DOI:** 10.5256/f1000research.24559.r62844

**Published:** 2020-05-11

**Authors:** Claudio Tiribelli

**Affiliations:** 1 Italian Liver Foundation, Trieste, Italy

This is an interesting study investigating in preterm neonates (PTNs) the predictive value of the bilirubin level assessed by transcutaneous technique (TcB) for hyperbilirubinemia (HB). 90 PTNs were and divided into two groups according to the weight (1500 g as diving value). Bilirubin was prospectively measured at different time points (12, 24, 48, and 72 h). Bilirubin level was confirmed by serum bilirubin measurement (TSB) if the TcB was “increased”. ROC curves were used to assess a 24 h TcB value predicting 48 and 72 h HB. Although the data may be of interest, the study suffers several intrinsic weaknesses what must be addressed before being considered further.

 Major Critiques:

1. The indication when TSB was measured is unclear and confusing. In the abstract it is stated that TSB was assessed before 72 h if TcB showed “abnormal results”. What does this mean? Detailed numerical values must be provided to allow to understand why TSB was performed.
2. On what basis the TSB value of ≥ 7 mg/dL in group 1 and > 10 mg/dL HB in group 2 was selected? This needs to be scientifically substantiated. At what time this level was measured?
3. How TSB was measured? Were the lab values confirmed by internal calibration? This must be clarified.
4. Fig 1 shows that the time course of HB is different in the 2 groups being the bilirubin peak reached 24 h later in group 2. This difference accounts for the different TcB cutoff values. This needs to be considered and addressed in the discussion.
5. How was the correlation between TcB and TSB at 72h? This information is important to assess the reliability of the two techniques (see also point #3).
6. The abstract is inaccurate as data reported are different from those indicated in the text (see for example the lack of 48H values).

Is the work clearly and accurately presented and does it cite the current literature?

No

If applicable, is the statistical analysis and its interpretation appropriate?

Partly

Are all the source data underlying the results available to ensure full reproducibility?

No

Is the study design appropriate and is the work technically sound?

No

Are the conclusions drawn adequately supported by the results?

Partly

Are sufficient details of methods and analysis provided to allow replication by others?

No

Reviewer Expertise:

Bilirubin, Jaundice

I confirm that I have read this submission and believe that I have an appropriate level of expertise to confirm that it is of an acceptable scientific standard, however I have significant reservations, as outlined above.

# Response

**Published:** 2020-06-25

**Authors:** Mahendra Tri Arif Sampurna

**Affiliations:** Faculty of Medicine Universitas Airlangga, Indonesia

**1). Reviewer's Comment:** The indication when TSB was measured is unclear and confusing. In the abstract it is stated that TSB was assessed before 72 h if TcB showed “abnormal results”. What does this mean? Detailed numerical values must be provided to allow to understand why TSB was performed.

**Author's response:**

 Dear Reviewer,

 Thank you for your comments.

 We added in our method à variables section as follows:

 The bilirubin level of each neonate was measured on the sternum by TcB (Dräger® Jaundice Meter 105) at 12 hours, 24 hours, and 72 hours with ±3 hours tolerance (the TcB measurement could be taken within three hours before/after the exact time). The TSB measurement was taken for each neonate at the age of three days or if the TcB bilirubin level was ≥5.76 (7-1.24)  mg/dL for Group I and TcB ≥8.76 (10-1.24) mg/dL for Group II and it had to be taken within six hours before or after the TcB measurement (assumption of TcB  standard deviation being ±1.24 mg/dL). The TSB measurement also had to be taken if the TcB measurement showed abnormal results. Hyperbilirubinemia was defined as TSB ≥7 mg/dL for preterm neonates with birth weights of 1000–1500 g and TSB >10 mg/dL for preterm neonates with birth weights of 1501–2000 g as suggested by the Kaplan et al., in Martin’s Neonatal-Perinatal Medicine (2011).

**2). Reviewer's Comment:** On what basis the TSB value of ≥ 7 mg/dL in group 1 and > 10 mg/dL HB in group 2 was selected? This needs to be scientifically substantiated. At what time this level was measured?

**Author's response:**

 Dear Reviewer,

 Thank you for your comments.

   

 The cut off 7 and 10 mg/dl are based on the recommendation of Martin Fanaroff (Reference source: Kaplan M, Wong R, Sibley E, et al.: Neonatal Jaundice and Liver Disease. In: Martin’s Neonatal-Perinatal Medicine. 2011; 1443–90.) In this recommendation they use birth weight category regardless postnatal age in hours.

**3). Reviewer's comment:** How TSB was measured? Were the lab values confirmed by internal calibration? This must be clarified.

**Author's response:**

 Dear Reviewer,

 Thank you for your comments.

 TSB measurements were performed by retrieving peripheral blood samples of each subject.

 Added in methods à variables section :

 Total serum bilirubin was measured in the central laboratory using SIEMENS Dimension® with a modified Doumas  22 reference method, which is a modification of the diazo method described by Jendrassik and Grof in 1938  23. Internal calibration was completed daily, with a quality control printout. The Indonesian External Quality Assurance Service performed external quality control.

**4). Reviewer's comment:** Fig 1 shows that the time course of HB is different in the 2 groups being the bilirubin peak reached 24 h later in group 2. This difference accounts for the different TcB cut-off values. This needs to be considered and addressed in the discussion.

**Author's comment:**

 Dear Reviewer,

 Thank you for the comment.

 We added in the discussion:

 It shows that that higher birth weight has opportunity to be hyperbilirubinemia later than the lower birthweight. The lower birthweight has lower threshold bilirubin, because they have higher chance to become encephalopathy in lower bilirubin level (high risk infants). The younger gestational age and lower birth weight, lead to a higher prevalence of infants developing hyperbilirubinemia. It is a result of excessive neonatal red cell hepatic and immaturity of the gastrointestinal system. Prematurely delivered infants have a likelihood of slower maturation of hepatic bilirubin uptake and conjugation.

**5). Reviewer's comment: **How was the correlation between TcB and TSB at 72h? This information is important to assess the reliability of the two techniques (see also point #3)

**Author's response:**

 Dear Reviewer, 

 Thank you for your comments.

 We actually did not assess the correlation between TcB and TSB at 72 hours of age. We would like to mention our reasons why we did not perform the assessment as the followings:

1. We were not specifically aiming at performing diagnostic test;
2. We only discovered fewer observed subjects who had reached observation at 72 hours of age thus the sample size at that age was not abundant in number. Most of the infants underwent phototherapy before 72 hours (group 1). Those infants were excluded from the study for further analysis.

**6) Reviewer's Comment: **The abstract is inaccurate as data reported are different from those indicated in the text (see for example the lack of 48H values).

**Author's response:**

 Dear Reviewer,

 Thank you for noticing. We adapted our abstract accordingly

 We also found that there was an unsuitable data between those mentioned in the study abstract and the ones in figures. However, we would like to inform that the proportional data provided in the study abstract is correct already meanwhile the ones in figures were attained by integrating the data percentages. Therefore, we are going to amend and adjust the data provided in abstract.
